# Improving Resident Comfort With Peripartum Cesarean Hysterectomy Through the Use of Low-Fidelity Simulation Models

**DOI:** 10.7759/cureus.69056

**Published:** 2024-09-10

**Authors:** Recia Frenn, Elise Heisler, Geoffrey Chen, Dani Zoorob

**Affiliations:** 1 Obstetrics and Gynecology, Loyola University Chicago Stritch School of Medicine, Chicago, USA; 2 Obstetrics and Gynecology, New York University, New York, USA; 3 Obstetrics and Gynecology, Adventist Health White Memorial, Los Angeles, USA; 4 Obstetrics and Gynecology, LSU Health Sciences Center in Shreveport, Shreveport, USA

**Keywords:** confidence, cost-effective, low-fidelity models, residents, simulation

## Abstract

Introduction

Cesarean hysterectomy is a relatively rarely performed, complex, life-saving procedure considered during post-partum hemorrhage and other obstetric complications. This multi-site study aimed at validating a low-cost, low-fidelity cesarean hysterectomy model to support resident proficiency and increase their confidence in performing this critical procedure.

Materials and methods

We developed a low-fidelity, anatomically representative model for cesarean hysterectomy simulation purposes. Obstetrics and Gynecology residents at two residency programs were offered a simulation experience using the model, with a performance assessment via two validated assessment tools. The clinical case involved a 38 y/o woman with a delivery complicated by postpartum hemorrhage who failed conservative therapy and required a peripartum hysterectomy. Participants included 26 residents, postgraduate year (PGY)1 through PGY4, at two midwestern university-based obstetrics and gynecology residency programs.

Results

The median resident scores on the Task Specific Checklist (TSC) and Global Rating Scale (GRS) correlated with increasing PGY levels. The combined TSC+GRS score was a median of 40 out of 49 total for the PGY4 class, while the PGY1 class had a median combined TSC+GRS score of 12 of 49. The PGY2 and -3 classes had TSC+GRS scores of 14 and 28, respectively. The simulation model was well-received with a median 4/5 rating for improving comfort level with cesarean hysterectomy and a median 4/5 rating for model realism.

Conclusion

This study validated a cost-effective, easily reproducible model that highlights the vital anatomy relevant to a cesarean hysterectomy. The model and simulation offer a way to introduce cesarean hysterectomies to residents while in training, particularly at sites that may not perform a substantial number of these procedures.

## Introduction

Cesarean hysterectomy is an uncommon, life-saving procedure considered during post-partum hemorrhage and following uterine atony, placenta accreta spectrum, or other obstetric complications [1]. Despite its general rarity, ranging between 0.20 to 5.09 per 1,000 deliveries, the incidence of emergency cesarean hysterectomies has increased over time, and the morbidity and mortality for both mother and newborn remain high [2]. For this reason, it is imperative that Obstetrics and Gynecology (OB/GYN) residents and attending physicians be specifically trained and prepared for such emergencies. However, a study in 2019 noted that among 417 US OB/GYN residents, only 21% reported having been primary surgeons during a cesarean hysterectomy, and 33% said they would not feel comfortable performing a cesarean hysterectomy post-residency [3]. This hesitancy from residents is not unique to the United States. A 2006 study from the UK found that only 44.6% of OB/GYN trainees would feel comfortable performing a caesarean hysterectomy when managing a major obstetric hemorrhage [4]. Similarly, a national survey of French OB/GYN residents found that 78% reported “not having the required skills” to perform an emergency peripartum hysterectomy [5].

Peripartum hysterectomy is a challenging procedure due to anatomical changes of pregnancy compounded by acute blood loss in a hemorrhaging patient. Simulation has been proven to increase the comfort, confidence, communication, and procedural skills of nurses and doctors in both emergency and non-emergent obstetric situations [6-9]. The use of three-dimensional models to simulate uterine atony has been shown to detect gaps in the knowledge and performance of OB/GYN residents, allowing program directors to improve training in specific areas of weakness [6]. Given the gravity of the context in which a cesarean hysterectomy is completed, in addition to the complexity of the procedure itself, the creation and use of a validated cesarean hysterectomy model is paramount in OB/GYN training and has not been published to date.

This study aims to use evaluation and assessment tools to validate a low-cost, low-fidelity cesarean hysterectomy model at multiple OB/GYN residency training sites. Additionally, we aim to increase resident comfort in performing this life-saving procedure through simulation.

## Materials and methods

Development and setting

The study received institutional board review exemption from both institutions. Obstetrics and Gynecology residents at two large midwestern university healthcare systems (Loyola University Medical Center and University of Toledo Medical Center) were offered the opportunity to simulate performing a cesarean hysterectomy on the model. The residents at both institutions completed the simulation late in the academic year during the same two-month time period (May and June 2022).

Literature was reviewed for cesarean hysterectomy education and simulation, and virtual meetings were held to address study details and model design. The focus was to ensure that the construct both reflected structures commonly tackled during the procedure and that they had a similar “feel” in the model-namely uterine vessels, anterior and posterior peritoneum, and the relation of the anterior uterus to the bladder and ureters. A low-fidelity model that closely reflects gravid pelvic anatomy was then developed for cesarean hysterectomy simulation purposes. The pelvic base for the model utilized a design from a previously published transvaginal hysterectomy simulation model which utilized a plastic pelvis mounted onto a wooden base [10].

Equipment/environment

The main equipment needed was the cesarean hysterectomy model (Video 1). In addition to the hysterectomy model, a standard hysterectomy tray (for surgical instruments) was utilized as were various sutures, including 0-Vicryl. The learner had access to surgical instruments that they would need as well as instruments that would not be necessary. The locations of the simulations were structured for convenience. They were performed in our office, boardrooms, or Labor and Delivery.

**Video 1:** Video with the assembly instructions for the cesarean hysterectomy model This video provides step-wise information about how to construct the TEACH model. This model will be used to perform the simulation assessment. TEACH: Trainee Enhancement of Ability in Caesarean Hysterectomy

Participants and personnel

Personnel included one learner (resident), one faculty instructor per model, and one assistant (typically a medical student). Eligibility criteria included affiliation with the authors’ accredited Obstetrics and Gynecology residency program, approval by the program leadership, and resident desire to participate in an educational study. Furthermore, residents were recruited based on the availability of their rotations during the study period. One residency program comprised 19 residents actively engaged in training, whereas the other consisted of 18 residents. Availability to participate in the study was limited to residents who were not on night shifts and those who were remote from reaching the maximum allowed ACGME (Accreditation Council for Graduate Medical Education) weekly hours. Residents were offered an opt-out option, if desired.

Implementation

Prior to the simulation, they were asked demographic questions, including number of cesarean hysterectomies and abdominal hysterectomies performed, as well as their confidence with performing cesarean hysterectomy independently (both on a 5-point Likert scale ranging from Strongly Disagree to Strongly Agree). The residents were provided with a case scenario (Table 1). At the start of the simulation, participants were oriented to the model components, the setup of the simulation, and expectations for performing a cesarean hysterectomy. The participants were asked to proceed with the simulation to the best of their ability and were allowed to end the simulation when they no longer knew the next surgical step. A uniform set of surgical instruments with both case-appropriate and inappropriate tools was made available for residents to choose from. Throughout the simulation, residents were asked to verbalize each step being performed. Residents were also provided with a surgical assistant who was instructed to only assist as instructed by the resident. They were advised to consider the assistant’s support as would be needed in real operating room situations.

**Table 1:** Simulation case for post partum hemorrhage requiring cesarean hysterectomy This imaginary case was developed to provide the resident with context regarding the situation. Alternative scenarios can be constructed based on institutional or program needs. TXA: Tranexamic acid

Brief Narrative Description of Case	This is a 39 years G8P8 who recently had a primary C/S after failed induction of labor for gestational diabetes. She had persistent uterine atony that has not responded to uterotonics. Decision was made to proceed with cesarean hysterectomy. Besides gestational diabetes, she is medically and surgically uncomplicated and her BMI is 26. Your task is to proceed with a cesarean hysterectomy to alleviate the uterine atony and postpartum hemorrhage. This simple model in front of you approximates a postpartum uterus and female pelvis for cesarean hysterectomy. I will ask you to perform a cesarean hysterectomy, stating clearly each step that you are going to be performing (state each next step planned). Please name the structures being addressed - if relevant. I cannot tell you the steps or what to do, but I can assist you if you need me to. I can also answer questions about the model if anything about the model isn’t clear. This setup has a full range of instruments and equipment which may assist you to perform the procedure. The patient is receiving appropriate blood product and IV fluid resuscitation as well as TXA and uterotonics. Sequential compression devices have been applied and activated. She has a foley catheter in place and the bladder is drained. Preoperative antibiotics have been administered. The patient has already been prepped and draped, and a time out has been performed. Please proceed with the surgery.
Primary Learning Objectives	Identify relevant anatomical landmarks involved in performing a cesarean hysterectomy. Identify appropriate surgical instruments and suture for cesarean hysterectomy. Demonstrate appropriate surgical assistance. Perform the necessary surgical steps of cesarean hysterectomy. Improve their comfort and confidence with performing cesarean hysterectomy.
Critical Actions	See cesarean hysterectomy Task Specific Checklist
Learner Preparation or Prework	No information should be given prior to the initiation of the case.

One faculty rater at each institution rated all the participants at their respective programs using the same two objective assessment tools. The faculty raters were not blinded. However, they were all trained to use the objective and standardized assessment tools. Prior to collecting this data, faculty members rated the same set of two non-resident simulation videos twice to confirm high inter- and intra-rater reliability.

Debriefing

Residents also answered a post-simulation survey regarding whether the simulation was realistic and if it improved comfort for performing cesarean hysterectomies in the future. The survey design was devised through a review of previously published literature [11-14]. Once the simulation was complete, the residents had the opportunity to debrief with the team on what went well and how cesarean hysterectomy simulation could be improved in the future (Table 2). 

**Table 2:** Debriefing guide This debriefing guide is used to assess subjective resident insight regarding the simulation. It focuses on their knowledge base and familiarity with the indications and variations in hysterectomy needs for postpartum hemorrhage cases.

Key Question	Points to Discuss
What are common risk factors for cesarean hysterectomy?	Similar risk factors for hemorrhage, atony and/or Placenta Accreta Spectrum (PAS): Grand multiparity Prior cesarean sections or other uterine surgery Placental abruption Infection Macrosomia Multiple gestation Extension of uterine incision at delivery PAS Cervical cancer, fibroids (often planned)
In what ways does a cesarean hysterectomy differ from a routine abdominal hysterectomy?	Distinguishing features of emergency hysterectomy on a pregnant patient: Greater vascularity: At term, the uterine blood flow increases to almost 1 L/min. This, in addition to engorged vessels that can occur if the patient was in labor, make the uterine vessels significantly increased in size. Proximity to ureters: Ureters are distended in pregnancy, not only due to gravid uterine compression but also due to progesterone effect on dilating ureteral smooth muscle. Propensity for coagulopathy: A known sequelae of cesarean hysterectomy includes Disseminated Intravascular Coagulation (DIC). Rapid blood loss can promptly spiral a patient into DIC. In contrast, postpartum patients are much more prone to blood clots. Supracervical hysterectomy: a subtotal hysterectomy is usually indicated as it is less likely to result in ureteral injury and can typically be completed more rapidly.
What are the key steps to completing the cesarean hysterectomy?	Identify round ligaments; suture, ligate and tag. Create a bladder flap Identify an avascular window in the broad ligament; open up the posterior and anterior leaf of broad ligament to allow ureter to be freely mobilized. Identify the utero-ovarian ligaments; clamp, cut, and suture/ligate. Open the broad ligament to skeletonize the uterine artery; clamp, cut, ligate and divide uterine vessels. Cuff closure.
What steps can be taken to ensure the patient is adequately resuscitated in the event of cesarean hysterectomy?	Ensure all uterotonics and/or pro-coagulants have been administered, specifically in cases of atony. Confirm methergine, hemabate, cytotec, tranexamic acid (TXA), etc. have been administered if indicated. Activate Massive Transfusion Protocol; ensure adequate resuscitation of blood and clotting factors if blood loss exceeds 1,500mL Monitor for signs and symptoms of impending DIC. This includes drawing hemorrhage labs (complete blood counts (CBC), type and screen, ABO grouping, basic metabolic panel (BMP), coagulation factors, fibrinogen +/- Thromboelastography (TEG) if available at institution) Closed loop communication. Clearly verbalize plan for cesarean hysterectomy. Ensure anesthesia is prepared for general anesthesia. Designate nursing to record Quantitative Blood Loss and which blood products have been administered.
What steps should be promptly considered after the hysterectomy is safely completed?	Redose antibiotics. Ancef 2g, if blood loss is > 1500mL or surgery > 4 hours, is always indicated Cystoscopy. Ensure the cystoscopy instruments and tower are nearby to evaluate for bladder injury. Keep the patient warm. Ensure blankets, bare-hugger, and blood product/crystalloids are infused to avoid hypothermia. Call ICU. Confirm that a surgical ICU bed is available for postoperative management. Disclose, document, and debrief with the patient and family.

Assessment

The two assessment tools used to evaluate resident performance using the model were the Task Specific Checklist (TSC) and the Global Rating Scale (GRS) (Tables 3, 4) [15-19]. The validated Global Rating Scale uses a scale of 1 to 5, with higher values representing more advanced skill, to evaluate respect for tissue handling, time and motion, instrument handling, knowledge of instrument selection, operation flow, assistant use, and familiarity with the procedure. The TSC was adapted from the previously published procedure-specific checklists for vaginal hysterectomy. It assesses residents on a “performed or not” basis regarding 14 surgical steps specific to cesarean hysterectomy.

**Table 3:** Task-specific checklist cesarean hysterectomy (assessment tool 1) This checklist focuses on the familiarity with steps needed to successfully and safely conduct a generic cesarean hysterectomy procedure.

		Yes	No
1	Appropriately sutures, ligates, and tags round ligaments		
2	Develops bladder flap		
3	Identifies an avascular window in the broad ligament		
4	Appropriately manipulates the uterus during the procedure		
5	Identifies utero-ovarian ligaments		
6	Appropriately clamps, cuts, suture/ligates utero-ovarian ligaments		
7	Appropriately dissects anterior/posterior leaf of broad ligament		
8	Identify the ureter		
9	Identifies uterine vessels		
10	Appropriately clamps, cuts, ligates, and divides uterine vessels		
11	Appropriately amputates the uterus from the cervix/vagina		
12	Appropriately closes cervical stump/vaginal cuff		
13	Assess for adequate hemostasis		
14	Assessment of bladder and ureters prior to closure		

**Table 4:** Surgical global rating scale (assessment tool 2) This table objectively assess the skillset of the resident conducting the simulation to help identify potential improvement opportunities. *Rating ranges between 1 to 5

Rating*	1	2	3	4	5
Respect for Tissue	Frequently used unnecessary force on tissue or caused damage by inappropriate use of instruments		Careful handling of tissue but occasionally caused inadvertent damage		Consistently handled tissue appropriately with minimal damage
Time and Motion	Makes unnecessary moves		Efficient time/motion but some unnecessary moves		Clear economy of movement and maximum efficiency
Instrument Handling	Repeatedly makes tentative or awkward moves with instruments by inappropriate use of instruments		Competent use of instruments but occasionally appeared stiff or awkward		Fluid moves with instruments and no awkwardness
Knowledge of Instruments	Frequently asked for wrong instrument or used inappropriate instrument		Knew names of most instruments and used appropriate instrument		Obviously familiar with the instruments and their names
Flow of Operation	Frequently stopped operating and seemed unsure if next move		Demonstrated some forward planning with reasonable progression of procedure		Obviously planned course of operation with effortless flow from one move to the next
Use of Assistants	Consistently placed assistants poorly or failed to use assistants		Appropriate use of assistant most of the time		Strategically used assistants to best advantage at all time
Knowledge of Specific Procedure	Deficient knowledge. Needed specific instruction at most steps		Knew all important steps of operation		Demonstrated familiarity with all aspects of operation

Statistical analysis

A descriptive analysis of surgical experience and resident characteristics was performed for each postgraduate year (PGY) class. It was hypothesized that higher scores on the TSC and GRS assessments would correlate with higher PGY levels, and they suggested increasing experience. Spearman correlation coefficients and their corresponding p-values were calculated to examine the correlation between GRS and TSC scores and years of training. All statistical tests were two-sided, and p-values <0.05 were considered statistically significant. All analyses were performed using SAS version 9.4 (SAS Institute Inc., Cary, USA).

## Results

Of the residents at both institutions, 26 of 37 (70%) met the eligibility criteria. All 26 (100%) residents participated and completed the cesarean hysterectomy simulation between May and June 2022. Whereas the median abdominal hysterectomies performed as a primary surgeon was 25 for the PGY4s in both residencies combined, one institution’s graduating class (PGY4, 2022) reported that they had not performed any cesarean hysterectomies during their residency while the other program reported performing an average of one. Prior to the simulation, all of the residents, including PGY4s, stated that they either disagreed or strongly disagreed with the statement “I feel confident performing cesarean hysterectomy independently.” Upon completion of the simulation exercise, the trainees reported appreciation of the simulation model with a median 4/5 rating for improving comfort level with cesarean hysterectomy and a median 4/5 rating for model realism (Table 5). The findings of this study were presented at the Central Association of Obstetricians and Gynecologists national meeting and received the multi-institutional excellence in research award (FAR award) in 2023.

**Table 5:** Resident assessment of simulation experience Residents were asked prior to the simulation: ^‘I feel confident to perform a cesarean hysterectomy independently’ Residents were asked after the simulation: *‘This simulation improved my comfort level for performing cesarean hysterectomies in the future’. #‘This cesarean hysterectomy model was a realistic teaching model for actual cesarean hysterectomies’. All three questions on a Likert scale 1-5, strongly disagree (1) to strongly agree (5) Q represents Quartiles (with Q1 representing the First Quartile, and Q3 representing the Third Quartile) PGY: postgraduate year

	All, N=27		PGY1, N=6		PGY 2, N=7		PGY 3, N=7		PGY 4, N=7	
	Median	Q1,Q3	Median	Q1,Q3	Median	Q1,Q3	Median	Q1,Q3	Median	Q1,Q3
Pre-sim surgical confidence^	1	1,2	1	1,1	1	1,1.5	1.5	1,2	2	1,2
Simulation improved surgical comfort*	4	4,5	4	4,5	4	4,5	4	4,5	4	4,5
Model realism#	4	4,5	4	4,5	4	4,5	4	4,5	4	4,5

**Figure 1:**
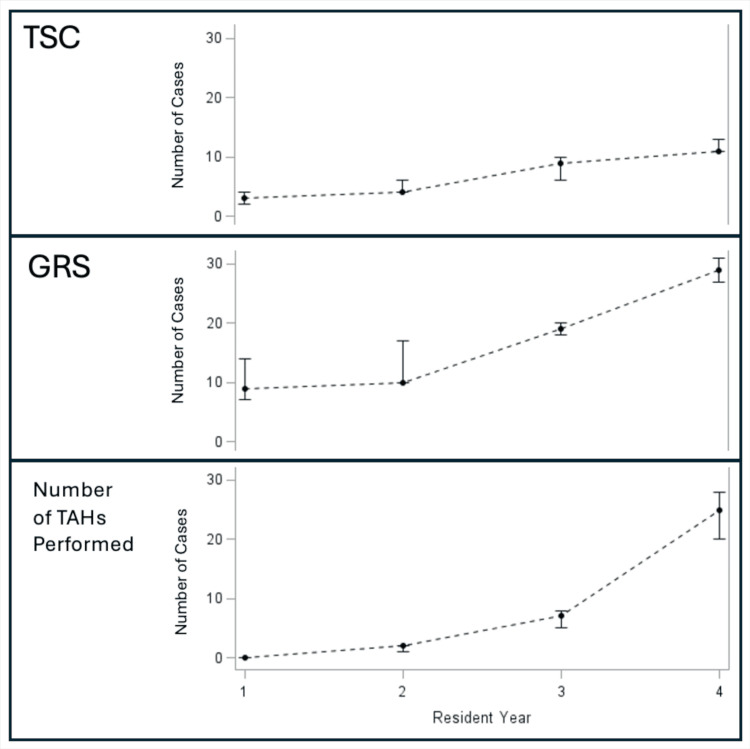
Correlation between TSC, GRS and PGY year An increase along the X-axis indicates the progression of each post-graduate residency year. An increase in numbers along the Y-Axis suggests an increase in cumulative score for the specific section. TSC - Task-Specific Checklist; GRS - Global Rating Scale; PGY - Postgraduate Year; TAH - total abdominal hysterectomy p<0.05, Significant

The median resident scores on the Task Specific Checklist and Global Rating Scale correlated with increasing PGY levels (Table 6 and Figure 1). The combined TSC+GRS score was a median of 40 out of 49 total for the PGY4 class, while the PGY1 class had a median combined TSC+GRS score of 12 of 49. The PGY2 and 3 classes had TSC+GRS scores of 14 and 28 respectively. 

**Table 6:** Performance assessment on model (median) TSC=Task Specific Checklist Total Score.  Maximum Score =14 GRS=Global Rating Scale Total Score.  Maximum Score=35 TSC+GRS=Combined Total Score. Maximum Score=49 PGY: postgraduate year

	PGY1, N=6	PGY2, N=7	PGY3, N=7	PGY4, N=7
TSC	3	5	7	10
GRS	9	10	19	28
TSC+GRS	12	14	28	40
Simulation time (min)	4	23	29	29

## Discussion

This study validated an easily reproducible and budget-conscious model that highlights the most important anatomy relevant to a cesarean hysterectomy. The model was developed, produced, and tested at two residency programs, with 26 residents participating in the study. Similar to other studies that evaluated resident performance through simulation, objective and standardized scoring on this simulation rose with advancing resident years. 

Despite cesarean hysterectomy being a life-saving procedure with increasing prevalence in the United States, exposure or experience during residency is not required for graduation. As a result, this could impact confidence in dealing with such a procedure independently post-graduation. Thus, gynecologic surgical educators from various residency programs aimed to develop this low-fidelity cesarean hysterectomy model to help fill this gap in education. 

**Figure 2:**
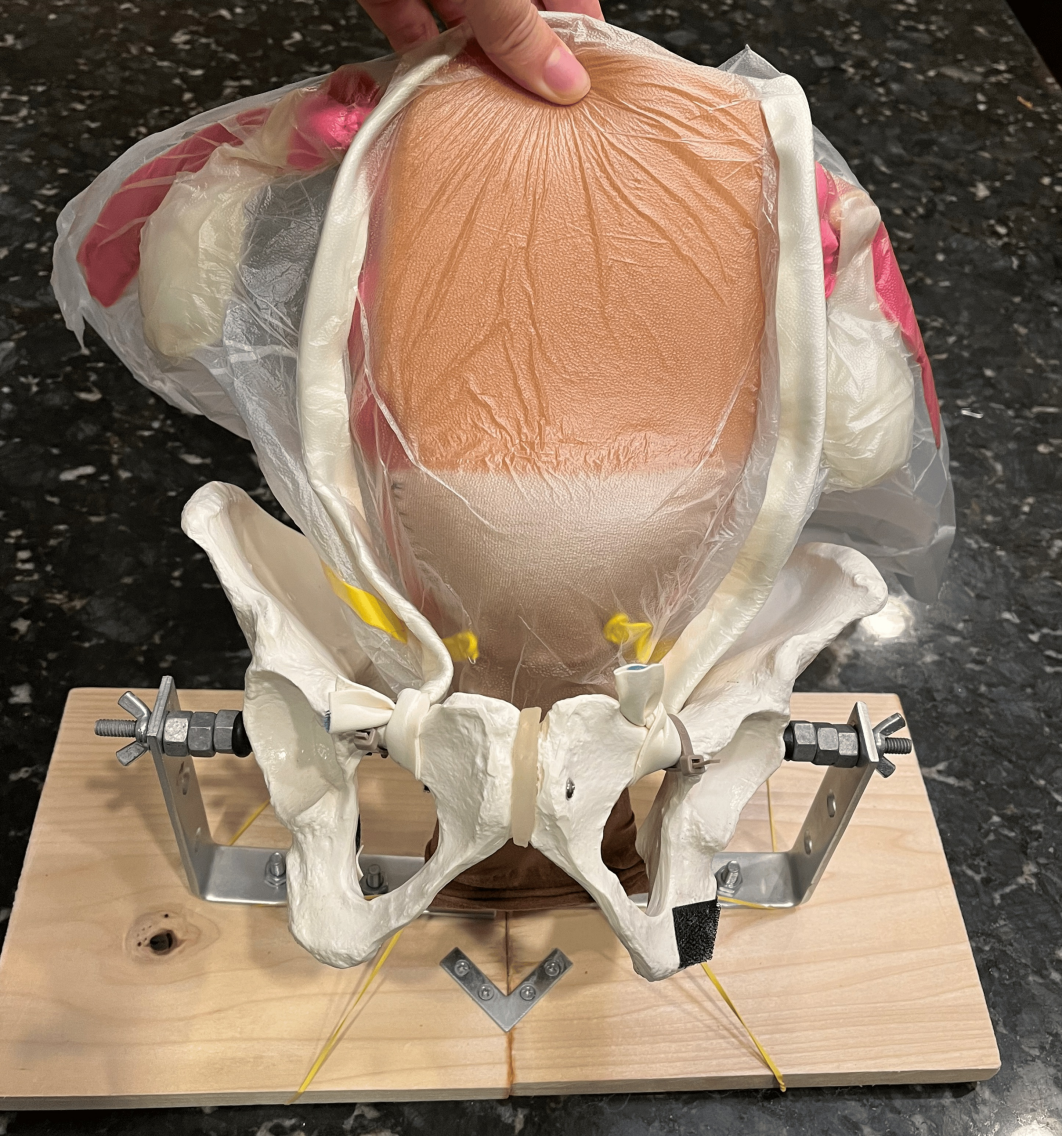
Completed model (front view)

**Figure 3:**
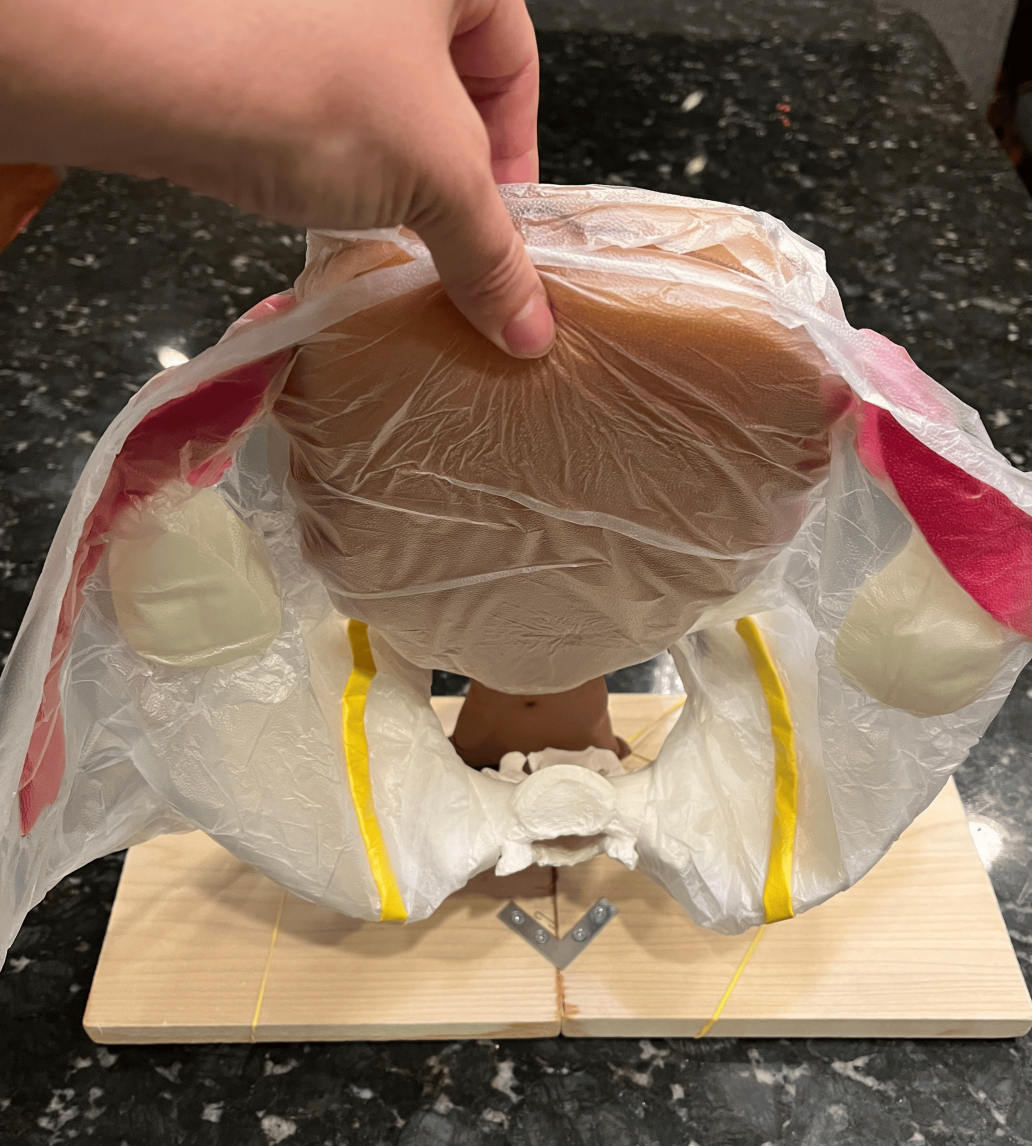
Completed model (top view)

This multisite study generated a realistic model with high reproducibility at a low cost (Figure 2 and Figure 3). To our knowledge, this is the first low-fidelity cesarean hysterectomy model that has been developed to correlate with all structures commonly involved in such a hysterectomy type, to be studied objectively across multiple institutions, and have a similar feel to real visceral structures while being budget-friendly. Thus, the model is a reliable and affordable way to introduce cesarean hysterectomies to residents while in training, particularly at sites that may not perform a substantial number of these procedures. 

Regarding cost-effectiveness, each uterine model cost $6.00 in material and it took less than one hour to build up to five models. Many components of the model, including the bladder, vagina, ovaries, and fallopian tubes were reusable. No additional skills were needed for the assembly of the uterine models. All materials used for construction were easily available online. Regarding the base and optional to the simulation was the use of the wooden base and pelvis. These cost approximately $50 to build for the first time and require around 2 hours to put together. Additionally, moderate carpentry familiarity may be needed. Alternatively, for simplicity and cost reduction, the simulation could also be completed without the pelvic base by securing the uterine model into a cardboard box.

Strengths and limitations

Limitations of this study included the fact that only 27 residents participated in the study from just two institutions. In addition, faculty evaluators were not blinded to the residents they rated. However, the training used did ensure that inter- and intra-rater reliability was adequate, and this is the largest study focused on assessing the useability of a model for cesarean hysterectomy. This model may also offer an objective assessment of resident skills and procedural knowledge, with ease of reproducibility of the model for subsequent simulations. The uterine model itself requires no special skills to build and is easy to produce in large quantities. Mounting the pelvis model onto the base does require drilling a hole through the model, which did pose an initial challenge, but once built was easily reusable for pelvic surgery simulation of multiple types. The simulation could be completed without the pelvic base if needed.

## Conclusions

This cesarean hysterectomy simulation model offers high anatomic similarity, ease of reproducibility, and construct validity to help residents achieve exposure to the procedure during training. Future studies may consider validating the simulation model on a larger scale with more residents at both academic and community-based residency programs. Although no classroom model can perfectly simulate the real-life experience of performing a cesarean hysterectomy, this model may help clinical educators introduce cesarean hysterectomy to their residents. In addition, it will help to raise comfort levels with cesarean hysterectomies, particularly for residents at programs that do not perform a high number of them. This could improve trainee confidence in performing this procedure independently. 
